# Development and Validation of a New Tool to Assess Burden of Dietary Sodium Restriction in Patients with Chronic Heart Failure: The BIRD Questionnaire

**DOI:** 10.3390/nu10101453

**Published:** 2018-10-07

**Authors:** Etienne Audureau, Aziz Guellich, Esther Guéry, Florence Canouï-Poitrine, Véronique Benedyga, Hélène Duchossoir, Charles Taieb, Thibaud Damy

**Affiliations:** 1 Public Health Department, CEpiA (EA4393), 94000 Créteil, France; etienne.audureau@aphp.fr (E.A.); esther.guery@gmail.com (E.G.); florence.canoui-poitrine@aphp.fr (F.C.-P.); 2 Heart Failure Unit, Department of Cardiology, INSERM U955, ARI, DHU ATVB, AP-HP Henri Mondor Hospital, University Paris Est Créteil (UPEC), 94000 Créteil, France; guelliaz@gmail.com; 3 Dietetic Unit, AP-HP Henri Mondor Hospital, University Paris Est Créteil (UPEC), 94000 Créteil, France; veronique.benedyga@aphp.fr (V.B.); helene.duchossoir@aphp.fr (H.D.); 4 EMMA, European Market Maintenance Assessment, 94080 Vincennes, France; charles.taieb@aphp.fr

**Keywords:** heart failure, questionnaire, sodium restriction, diet, burden

## Abstract

(1) Background: Burden scales are useful in estimating the impact of interventions from patients’ perspectives. This is overlooked in sodium diet/heart failure (HF). The aim of this study is to develop and validate a specific tool to assess the burden associated with low-sodium diets in HF: the Burden scale In Restricted Diets (BIRD). (2) Methods: Based on the literature and reports from patients, 14 candidate items were identified for the following dietary-related domains: organization, pleasure, leisure, social life, vitality, and self-rated health. The validation study was conducted prospectively. The questionnaire was refined via item reduction according to inter-item correlations and exploratory factor analysis. Internal consistency was determined using Cronbach’s alpha (Cα) and convergent validity by assessing correlations between BIRD and the health-related quality of life (HRQoL) Minnesota Living with HF questionnaire (MLHF). (3) Results: Of the 152 invited patients, 96 (63%) returned the questionnaire. The median score was 6.5 (IQR 2.0–14.0). The results showed good acceptability (non-response rates/item from 2.0% to 12.1%), an excellent internal consistency (Cα = 0.903) and a good convergent validity (rhos = 0.37 (physical), 0.4 (mental), and 0.45 (global); all *p* < 0.05). (4) Conclusions: BIRD demonstrates good psychometric properties and is useful to quantify the burden associated with sodium restriction. It may help optimize dietary interventions and improve the overall management of patients with HF.

## 1. Introduction

Chronic heart failure (HF) is a major public health and social problem. Its prevalence increases with age, reaching approximately 10% after 70 years [1,2]. This high prevalence is expected to evolve further as a result of the continuous aging of the population, improved survival of patients with different heart diseases, and effective treatments for HF. Despite the significant progress in the diagnosis and treatment of HF, this condition remains a major cause of morbidity and mortality, with a 5-year mortality rate of ~50% after the first onset of symptoms [3]. Management of HF includes both pharmacological and non-pharmacological interventions. Among non-pharmacological interventions, dietary sodium restriction is commonly recommended and is endorsed by most international guidelines [4,5]. However, these recommendations are based on limited evidence with inconsistent findings across studies [6]. Furthermore, available data suggest that less than half of patients with HF actually follow these recommendations [7], raising questions about compliance and the underlying reasons for non-compliance.

Compliance to diet is closely related to patients’ subjective perceptions and expectations. In this regard, chronic heart failure is a complex condition that requires important personal investment from patients to manage their disease. However, the alteration in their long-standing personal habits and lifestyle may lead to their perception of the dietary sodium restriction diet as a negative intervention [8]. Indeed, many patients with HF may face a variety of constraints due to this regimen that cover the social, emotional, organizational, and economic aspects of daily life—all limitations that can be captured by the notion of “burden”.

The current existing scales for evaluating quality of life in patients with HF are fundamentally limited for assessing this burden, since they include few to no items inquiring about a patients’ diet, whether they be generic, like the SF-36 [9]; disease-specific, like the Minnesota living with heart failure questionnaire [10]; or diet-targeted but focused on conditions too specific to be generalizable to other contexts [11,12].

To the best of our knowledge, there is no tool available for evaluating the burden experienced by patients with heart failure on a low-sodium diet, so there is a pressing need for accurate tools to measure this burden. Thus, developing a dedicated instrument would be beneficial to ascertain the concerns of HF patients’ and their physicians alike for identifying which patients are in need of more sustained monitoring or support for their diet. The objective of the present study, is therefore, to develop and validate a new scale—the “Burden scale In Restricted Diets” (BIRD)—to allow an adequate evaluation of the burden associated with dietary sodium restriction in heart failure patients.

## 2. Methods

The BIRD questionnaire was designed based on standardized health care, health-related quality of life (HRQoL) questionnaire development, and validation methodology [13,14]. A multidisciplinary team was set up to ensure the scientific and clinical relevance of the process, including cardiologists, dietitian nutritionists, and experts in questionnaire conception and quality-of-life indexes.

### 2.1. Development

The first stage included the creation of a verbatim report based on review of the literature, and qualitative information collected during semi-/unstructured interviews with patients with HF to discuss their complaints and distresses related to diet in HF. Based on this report and input from the multidisciplinary working group, the major identified concerns were meal preparation, pleasure, budget, leisure, social life, consequences at work, vitality, and self-rated health.

An initial set of 14 candidate items were produced and grouped according to their content to constitute the initial draft questionnaire (Table 1). Wording and response modalities were tested in native French-speaking subjects during individual interviews to determine potential issues (ambiguity, misunderstanding, acceptability). A five-point Likert scale was used (not at all, just a little, somewhat, quite a lot, very much) with answers numbered from 0 to 4 accordingly. To limit missing data, the modality ‘Not applicable’ was also included for items 4, 5, 7, 9, and 12 related to meals away from home, grocery shopping, close relatives preparing meals, leisure activities, and professional activity, respectively. A global score was calculated by summing individual item scores, where a higher total score represents a higher symptom burden.

### 2.2. Validation Study

A validation study was conducted to assess the psychometric properties of the scale. A cross-sectional survey was conducted in more than 100 adult patients with medically diagnosed HF using the draft questionnaire. The questionnaire was pretested in the first 10 patients to evaluate the comprehensibility, and changes were made based on their comments. To be eligible, patients had to be able to read, be able to understand and speak the French language, and lack any cognitive impairment. Patients were prospectively enrolled in the Cardiology Department of the Henri-Mondor Hospital, Creteil, France. Given the nature of the study, without any change to patient care, the need for written informed consent was waived under French regulations; all patients received an information sheet with contact details, and authorization to use data could be withdrawn at any time. The study protocol conforms to the ethical guidelines of the 1975 Declaration of Helsinki.

### 2.3. Statistical Analysis

Psychometric analysis included assessment of item characteristics, construct validity, internal consistency, known-groups validity, and convergent validity.

Descriptive analyses were performed to study the distribution of individual items and global score, to inform on the acceptability (% missing values), and to identify potential ceiling and/or floor effects when a majority of item responses were distributed at either end of the scale. Spearman’s rank correlation coefficients (r_s_) were computed to examine the homogeneity of the scale (item-total correlation, with a minimal acceptable level of r_s_ ≥ 0.3) and to identify whether highly correlated items should be omitted for redundancy (inter-item correlations, r_s_ > 0.8). A correlation network plot was built from those results to graphically illustrate relationships. A hot-deck multiple imputation was performed to impute missing data for subsequent analyses.

Construct validity (factor structure) of the instrument was assessed through exploratory factor analysis (principal factor method) to examine the underlying constructs and characterize the scale dimensionality. Data were first examined with the Bartlett’s test of sphericity (*p* < 0.0001) and the Kaiser–Meyer–Olkin measure of sampling adequacy (0.842), indicating that our sample was suitable for conducting exploratory factor analyses (EFAs). As recommended [15,16], we used a combination of various strategies to determine the optimal number of factors to retain, including consideration of the proportion of variance explained for the factor solution retained, using Horn’s parallel analysis [17] based on the 95th percentile estimate and computing Velicer’s minimum average partial (MAP) criterion [18]. Items were considered for deletion if their factor loadings were <0.4, or/and if their communalities were <0.3 (uniqueness > 0.7).

Internal consistency reliability (homogeneity of the items) was assessed by calculating Cronbach’s alpha [19]. A coefficient score of >0.8 indicates good internal consistency and >0.9 is considered an excellent one.

Known-groups validity was investigated by studying whether the burden score would differentiate between adjacent severity subgroups. For that purpose, differences in burden score were assessed according to the New York Heart Association (NYHA) class and the intensity of the prescribed low-sodium diet (normal: >6 g per day; moderately restricted: 3–6 g; highly restricted: <3 g). Scores were compared across groups using the nonparametric Kruskal–Wallis test for global comparisons and the Jonckheere–Terpstra test for trends.

Convergent validity was studied by assessing correlations between the global burden score and the HRQoL Minnesota Living with Heart Failure questionnaire (MLHF), a validated 21-item questionnaire for assessing HRQoL in patients with HF [10]. Spearman’s rank correlation coefficients were computed between the burden score and each MLHF domain score, namely, physical (8 items, score 0–40), emotional (5 items, 0–25), and other items (8 items, 0–40). Convergent validity measures how closely related the BIRD score is to other scales measuring similar but not strictly equivalent constructs. Coefficients less than 0.3 were considered as weak, from 0.3 to 0.5 as moderate, and those above 0.5 as strong.

Statistical analyses were performed using Stata v14.1 software (StataCorp, College Station, TX, USA) for descriptive and factor analyses and R 3.2.4 software (R Foundation for Statistical Computing, Vienna, Austria) using the *hot.deck*, *paran*, and *psych* packages for hot-deck imputation, Horn’s parallel analysis, and Velicer minimum average partial correlation for number of principal components, respectively.

### 2.4. Translation and Cross-Cultural Validation

Since the original BIRD questionnaire was developed in French, a well-established methodology was applied to generate an English version. This included forward and backward translation while accounting for cross-cultural validation [20]. Linguistic and cross-cultural adaptation was carried out by a specialized institution (Lionbridge, Dublin, Ireland) following a nine-step process for the English language (Supplemental Table S1).

## 3. Results

Of the 152 patients invited, 96 (63%) completed and returned the questionnaire. Comparison between non-participating and participating patients for age, gender, and NYHA class did not reveal any significant differences (data not shown). Main characteristics of the sample, including sociodemographics, clinical and biological features, HF-related diet treatment, and Minnesota Living with Heart Failure (MLHF) scores by domains are shown in Supplemental Table S2. The sample included 72% men with a mean age of 62 ± 12 years, of whom 26% had a highly restricted low-sodium diet (<3 g/day).

### 3.1. Item Statistics

**Figure 1:**
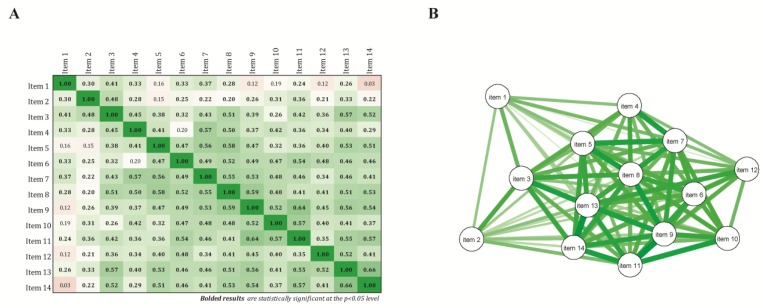
Correlation between items: (**A**) Spearman’s rank correlation coefficients matrix and (**B**) correlation network. The matrix contains the Spearman’s rank correlation coefficients between the 14 items of the candidate questionnaire. Colors indicate the direction and the strength of the correlation, with positive correlations being displayed as green tones and negative ones as red tones. Bolded results indicate statistical significance at the *p* < 0.05 level. The correlation network is constructed from all pairwise correlations between items in (**A**). Items are represented by nodes and are connected by edges. Red and green lines represent negative and positive correlations, respectively. Line width color saturation is proportional to the strength of the correlation.

Of the 96 participating patients, 69 (72%) answered all the questionnaire items. Among those who did not, missing data ranged from 1 (17%) to 5 (1%) of the items. Item statistics related to data quality, distribution, and mean scores are shown in Table 2. Mean scores by item varied between 0.6 (items 3, 12, and 14) and 1.7 (item 1), with floor effects reached in items 2, 3, 5, 10, 13, and 14 (>50% of the responses at the lowest end of the scale). There was no evidence of ceiling effects. Missing data ranged from 1% to 10% (item 12, related to professional activity), while non-applicable answers varied between 4% and 27% (item 12) for the four items including this answer option. Spearman correlation coefficients between items and the total score were all above 0.6, with the exception of items 1 (r_s_ = 0.47), 12 (r_s_ = 0.53), and 2 (r_s_ = 0.55). Lower inter-item correlations were apparent for items 1, 2, and, to a lesser extent, 12 (Figure 1). No inter-item correlation exceeded 0.8, thus ruling out potential redundancy at this stage.

### 3.2. Factor Structure

A one-factor solution explained 79.8% of the total variance, with both the MAP criterion and parallel analysis supporting this solution. The unadjusted and adjusted eigenvalues (parallel analysis) for the first three factors were 5.93/0.78/0.53 and 4.87/−0.04/−0.12, respectively. Items 1 and 2 were left out because of their insufficient loading (<0.4) and/or communality (<0.3) on the first axis. Factor analysis rerun after exclusion of these two items confirmed the scale unidimensionality, supported by the MAP criterion, parallel analysis, and the large proportion of total variance (88.0%) being explained by the first factor. These findings indicate construct validity and thus validate the calculation of a single BIRD score, which brings together all the items. A global 12-item burden score was then computed (maximum score: 48; mean: 9.8 ± 10.0; median: 6.5 (IQR 2–14) (Supplemental Table S3). The complete distribution is shown in Supplemental Figure S1.

### 3.3. Internal Consistency, Convergent and Known-Groups Validity

**Figure 2:**
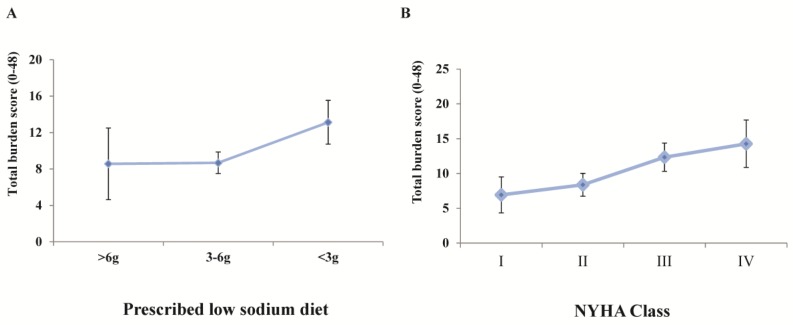
Global 12-item BIRD score according to (**A**) prescribed low-sodium diet and (**B**) NYHA class. *Error margins indicate standard error of the mean (SEM). NYHA: New York Heart Association.*

The developed scale showed excellent internal consistency (Cronbach alpha = 0.912). Results for known-groups validity are shown in Figure 2. As expected, patients with an increased NYHA class scored significantly higher on the global score (global test *p* = 0.055; test for trend *p* = 0.003). This supports the scale’s ability to discriminate according to clinical disease severity. A statistical trend for higher scores was also found in patients with the most restricted diet (global test *p* = 0.30; test for trend *p* = 0.06; (<3 g) vs. (3–6 g or >6 g) *p* = 0.06), supporting the ability of the scale to identify those who suffer the heaviest burden of a low-sodium diet. Table 3 exhibits the convergent validity results. Significant positive correlations were found between the global burden score and scores from the MLHF scale domains and global score: r_s_ = 0.37 (physical domain), 0.40 (mental domain), 0.45 (global score); all *p* < 0.05.

## 4. Discussion

Health-related quality of life (HRQoL) and burden scales have proved to be useful in estimating the impact of diseases and treatments from the patient’s perspective on their physical, mental, and social health status. Several generic or disease-specific patient-reported outcomes are available to assess the self-rated health status of patients with heart failure. However, none capture the specific domains related to the sodium restriction often prescribed in HF. To the best of our knowledge, BIRD is the first validated questionnaire assessing specifically the burden of a low-sodium diet in patients with HF. BIRD was found to be psychometrically robust, with excellent internal consistency and good item-scale, convergent validity, and construct validity. BIRD also correlated significantly with components of the MHF, confirming its concurrent validity.

### 4.1. Dietary Sodium Restriction and Quality of Life in HF

Heart failure is associated with activation of the neurohormonal system, leading to sodium and water retention. All national and international guidelines currently recommend that HF management should include dietary sodium restriction [21]. Based on pathophysiological and experimental studies, it has been assumed that a low-sodium diet would relieve symptoms and improve the quality of life and outcomes in HF patients. However, observational and clinical studies have shown mixed results [6]. Although adverse neurohormonal activation related to sodium restriction in HF remains a concern, the benefit-impact balance for patients’ HRQoL with regard to sodium restriction in this context is unknown, with a substantial gap in the ability to evaluate the impact of this regimen. The low-sodium diet effects on quality of life have only so far been considered a potential favorable outcome, despite the fact that the burden related to restricted diets has extensively been described and characterized in several other diseases. It has thus been reported in children and to a lesser extent in adults for chronic diseases, including celiac disease, irritable bowel syndrome, and diabetes [22,23,24,25,26,27]. Data regarding sodium restriction in HF are missing, essentially because of the lack of a validated and accurate measurement tool.

### 4.2. Burden in Chronic Diseases and HF

The notion of ‘burden’ has been introduced by the World Health Organization to help quantify the health of a population and to determine the priorities for public health interventions [28]. The ‘burden of disease’ concept has been extended to now distinguish between (i) the overall burden, by measuring the economic impact on society, and (ii) the individual burden, relating to patients and their families. The latter assesses disability in a broad sense, i.e., psychological, social, economic, and physical, also accounting for everyday-life organization and the use of medical resources. The concept of burden has increasingly been used in medical research since it provides useful insight for evaluating the care of chronic diseases. In the context of HF, such burden may relate to a variety of accumulating constraints [8]. Patients are required to follow complex medication regimens and to implement important lifestyle changes, such as sodium restriction. Combined with the direct consequences of the disease, treatment burden may contribute to increasing the patient’s stress and decreasing their adherence to treatment.

### 4.3. Implications for Practice

BIRD is a quick and easy-to-use tool allowing a single diet-related burden score to be calculated. It has the advantage of covering multiple and relevant aspects of the burden that can result from a low-sodium diet in a patient’s daily life. Its widespread administration will be facilitated by the aptness of the questionnaire, as demonstrated by the satisfying psychometric properties of the instrument in the present study, i.e., understandability, reliability, and validity, as well as by its easy-to-use and parsimonious structure, with only 12 items to collect. As such, it may serve as a strategic tool for screening patients suffering the highest burden from their diet and who might benefit from specific interventions, such as psychological and social help, close follow-up, etc. It may also foster negotiation and cooperation between patients, physicians, health authorities, and agro-alimentary industries by providing a common tool for quantifying diet-related constraints. Finally, it is noteworthy that the items constituting the BIRD questionnaire relate to organizational, social, and general health status aspects which would most likely also be affected in other conditions requiring restricted diets. Thus, it would be of great interest to study the validity and clinical utility of the instrument in other settings and for other conditions, e.g., diabetes and celiac disease, and, ultimately, to compare the resulting burden scores using this common reference scale.

### 4.4. Limitations

Limitations associated with this study include the monocentric recruitment and the relatively limited sample size that restrained the possibility of subgroup analyses. Larger confirmatory studies using the BIRD questionnaire are warranted to further explore the potential effects of age, disease onset, culinary culture and habits, comorbidities, coexistence of other diet and restrictions, etc. on the burden of sodium restriction in HF. Second, 37% of invited patients did not complete and return the questionnaire. Yet, our response rate appears consistent with, and even somewhat higher than, those generally observed in surveys where participants are required to send questionnaires by postal mail, as in the present study. Relatedly, a selection bias resulting from such non-participation cannot be ruled out, though likely of limited magnitude, as there was no significant difference in demographics and NYHA classes between participating and non-participating patients. Finally, due to the cross-sectional design of the study, we also lacked the ability to investigate the predictive value of the tool for future treatment compliance and/or subsequent cardiologic events. Further research efforts will include the conduct of prospective studies to address these elements.

## 5. Conclusions

In conclusion, there is a need for physicians to recognize the individual burden of patients with HF under sodium restriction. Quantification of this burden by the BIRD questionnaire may help optimize dietary interventions and improve the overall management of patients with HF.

## Figures and Tables

**Table 1:** Initial candidate items for the “Burden scale In Restricted Diets” (BIRD) questionnaire.

On Account of My Diet, I am Not Living AS I Would Like, Because…	
**Item 1**	…I have to limit my consumption of my favorite dishes
**Item 2**	…my appetite is decreased
**Item 3**	…every meal is difficult for me
**Item 4**	…having a meal away from home is complicated
**Item 5**	…grocery shopping is complicated
**Item 6**	…it results in additional expenses
**Item 7**	…I have the impression of being a bother or a burden to those preparing my meals
**Item 8**	…it makes relationships or activities with friends or family difficult
**Item 9**	…it makes my leisure activities difficult (favorite pastimes, sports)
**Item 10**	…it prevents me from travelling, going on vacation
**Item 11**	…it makes me feel tired, weary or I lack energy
**Item 12**	…it is difficult to manage in my workplace/professional activity
**Item 13**	…it depresses me
**Item 14**	…it aggravates my health

**Table 2:** Item distribution and item-total score correlations.

	Mean ± SD	Item-Total Score Correlation	Item Distribution							Missing Data

			Total	Not at All 0	Just a Little 1	Somewhat 2	Quite a Lot 3	Very Much 4	Not Applicable	
**Item 1**	1.7 (±1.1)	0.47	94	16 (17%)	27 (29%)	27 (29%)	18 (19%)	6 (6%)	0 (0%)	2 (2%)
**Item 2**	1.0 (±1.1)	0.55	93	40 (43%)	24 (26%)	19 (20%)	7 (8%)	3 (3%)	0 (0%)	3 (3%)
**Item 3**	0.6 (±1.0)	0.66	95	65 (68%)	15 (16%)	8 (8%)	4 (4%)	3 (3%)	0 (0%)	1 (1%)
**Item 4**	1.1 (±1.2)	0.60	95	40 (42%)	19 (20%)	15 (16%)	13 (14%)	4 (4%)	4 (4%)	1 (1%)
**Item 5**	0.8 (±1.1)	0.61	94	48 (51%)	19 (20%)	7 (7%)	12 (13%)	2 (2%)	6 (6%)	2 (2%)
**Item 6**	0.8 (±1.1)	0.67	89	50 (56%)	21 (24%)	8 (9%)	7 (8%)	3 (3%)	0 (0%)	7 (7%)
**Item 7**	0.8 (±1.1)	0.69	95	44 (46%)	19 (20%)	11 (12%)	11 (12%)	1 (1%)	9 (9%)	1 (1%)
**Item 8**	0.7 (±1.0)	0.70	90	52 (58%)	19 (21%)	13 (14%)	3 (3%)	3 (3%)	0 (0%)	6 (6%)
**Item 9**	1.1 (±1.5)	0.71	91	43 (47%)	14 (15%)	8 (9%)	8 (9%)	12 (13%)	6 (7%)	5 (5%)
**Item 10**	0.8 (±1.2)	0.66	90	52 (58%)	16 (18%)	7 (8%)	11 (12%)	4 (4%)	0 (0%)	6 (6%)
**Item 11**	1.3 (±1.3)	0.78	94	35 (37%)	27 (29%)	12 (13%)	12 (13%)	8 (9%)	0 (0%)	2 (2%)
**Item 12**	0.6 (±1.1)	0.53	86	38 (44%)	9 (10%)	7 (8%)	6 (7%)	3 (3%)	23 (27%)	10 (10%)
**Item 13**	0.7 (±1.2)	0.71	94	61 (65%)	12 (13%)	12 (13%)	3 (3%)	6 (6%)	0 (0%)	2 (2%)
**Item 14**	0.6 (±1.0)	0.64	93	61 (66%)	17 (18%)	7 (8%)	5 (5%)	3 (3%)	0 (0%)	3 (3%)

*SD: standard deviation.*

**Table 3:** Results for convergent validity: Spearman correlation coefficients between the global burden score and the Minnesota Living with Heart Failure questionnaire domains.

	Minnesota Living with Heart Failure			
	Physical Domain	Emotional Domain	Other Items	Global Score
*Minnesota Living with Heart Failure*				
**Physical Domain**				
**Emotional Domain**	0.80			
**Other Items**	0.78	0.83		
**Global Score**	0.94	0.92	0.93	
**Global Burden Score**	0.37	0.40	0.44	0.45

*All results are statistically significant at the p < 0.05 level.*

## Supplementary Materials

The following are available online at http://www.mdpi.com/2072-6643/10/10/1453/s1, Figure S1: Global 12-item BIRD score distribution in the validation study population, Table S1: Steps for the linguistic and cross-cultural adaptation, Table S2: General characteristics of the study population, Table S3: One-factor solution of the BIRD questionnaire: results for the initial 14-items and after deletion of items 1 and 2.

## Abbreviations

**HF**	chronic heart failure
**BIRD**	Burden scale In Restricted Diet
**HRQoL**	health-related quality of life
**EFA**	exploratory factor analyses
**MAP**	minimum average partial
**NYHA**	New York Heart Association
**MLHF**	Minnesota Living with Heart Failure questionnaire
