# Supplementary material for: Development and Validation of a New Tool to Assess Burden of Dietary Sodium Restriction in Patients with Chronic Heart Failure: The BIRD Questionnaire
Source: Nutrients. 2018 Oct 7;10(10):1453. doi: 10.3390/nu10101453 (PMC6213449; doi:10.3390/nu10101453)
Supplement: Supplementary file 1 [file nutrients-10-01453-s001.pdf]

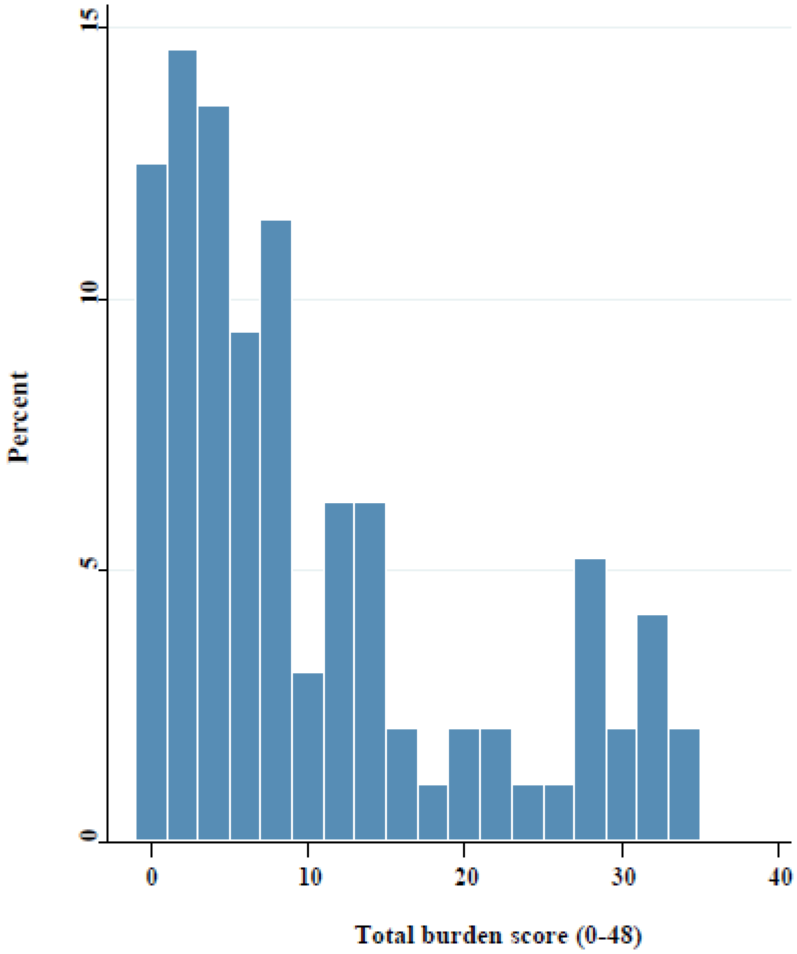

Figure S1. Global 12-item BIRD score distribution in the validation studypopulation (N=96).

Table S1. Steps for the linguistic and cross-cultural adaptation.

**Step**		**Details**
1	Preparation	Evaluation of the source text from a linguistic and cultural point of view including definition of concepts
2	Forward translations	Forward translation into English by two independent translators
3	Reconciliation	Comparison of the two forward translations to provide the best adaptation and produce a draft version of the text
4	Back translation	Translation of the draft forward translation back into English without reference to the original language
5	Back-translation review	Comparison of the original text and the back translation to verify that the meaning of the draft translation is equivalent to source
6	Analysis and implementation of back-translation review report	Analysis of the back-translation review report to verify if there are changes required to the draft forward
7	Pilot testing	Clinical review and cognitive debriefing

8	Review of cognitive debriefing or clinical review results	Review of the results from the cognitive debriefing or clinical review to identify translation modifications necessary for improvement
9	Proofreading and finalization	Last stage, which aims to a cross-cultural and validated translation of the questionnaire

Table S2. General characteristics of the study population (N=96).

Variable		N (%)
**Gender, women**		27 (28.1)
**Age, years**	Mean ±SD	62.4 ±12.3
	<55 years	26 (27.1)
	55-70 years	42 (43.8)
	>70 years	28 (29.2)
**Hypertension (%)**		43 (49.4%)
**Diabetes (%)**		24 (27.6%)
**Obesity (%)**		19 (21.8%)
**Body Mass Index, kg/m²**	Mean ±SD	26.5 ±5.4
**NYHA classification**	I	14 (17.5)
	II	29 (36.3)
	III	26 (32.5)
	IV	11 (13.8)
**Left ventricular ejection fraction, %**	Median (IQR)	31.5 (25.0;45.0)
**NT-proBNP, pg/ml**	Median (IQR)	1480.0 (452.0;3290.0)
**Serum creatinine, umol/L**	Median (IQR)	97.0 (77.0;130.0)
**Prescribed low sodium diet**	Normal >6 g	7 (8.0)
	Moderately restricted 3-6 g	57 (65.5)
	Highly restricted <3 g	23 (26.4)
**Minnesota Living with Heart Failure**		
**Total score (0-105)**	Mean ±SD	36.8 ±26.0
**Physical domain (0-40)**	Mean ±SD	16.1 ±11.6
**Emotional domain (0-25)**	Mean ±SD	8.0 ±6.9
**Other items (0-40)**	Mean ±SD	12.7 ±9.7

Results are N (%) unless otherwise stated. SD: Standard deviation; NYHA: New York Heart Association; IQR: Interquartile range.

Table S3. One-factor solution of the BIRD questionnaire: results for the initial 14-items and after deletion of items 1 and 2.

	14-items		*12-items*	
**Item**	**Factor loadings**	**Communality values**	**Factor loadings**	**Communality values**
**1**	0.37	0.14	-	-
**2**	0.42	0.17	-	-
**3**	0.66	0.44	0.62	0.39
**4**	0.59	0.35	0.58	0.34
**5**	0.66	0.43	0.67	0.45
**6**	0.67	0.45	0.66	0.44
**7**	0.71	0.51	0.71	0.50
**8**	0.75	0.56	0.75	0.57
**9**	0.74	0.55	0.75	0.57
**10**	0.63	0.40	0.63	0.40
**11**	0.72	0.52	0.72	0.51
**12**	0.58	0.34	0.59	0.35
**13**	0.76	0.58	0.76	0.58
**14**	0.70	0.50	0.72	0.52
